# Prevalence and Impact of Comorbid Cancer and Dementia on Health Outcomes in Older Adults: A Longitudinal Study

**DOI:** 10.1093/geroni/igab046.694

**Published:** 2021-12-17

**Authors:** Jyotsana Parajuli, Diane Berish, Ying-Ling Jao, Yo-Jen Liao, Lee Ann Johnson

**Affiliations:** 1 Univerisity of North Carolina at Charlotte, Charlotte, North Carolina, United States; 2 Pennsylvania State University, University Park, Pennsylvania, United States; 3 Penn State University, University Park, Pennsylvania, United States; 4 Univeristy of Virginia, University of Virginia, Virginia, United States

## Abstract

Dementia and cancer are two common chronic conditions in older adults. However, there are few studies examining the prevalence of comorbid cancer and dementia and the longitudinal impact of these comorbid conditions on health outcomes. This study investigated the prevalence and longitudinal impact on health outcomes in older adults with comorbid cancer and dementia. This is a secondary analysis, using data from the 2010 and 2016 waves of the Health and Retirement Study (HRS). The health outcomes of the study included nursing home stay, hospital stay, home care use, activities of daily living (ADL) limitations, instrumental activities of daily living (IADL), self-rated health status, mortality, and the out-of-pocket medical expenditure in older adults with cancer and dementia. Data were analyzed using descriptive statistics, logistic regression, and linear regression analyses. The results revealed that the prevalence of comorbid cancer and dementia ranged from 2.6% to 2.8% over the 6-year period. Older adults with comorbid cancer and dementia demonstrated higher likelihood of nursing home stay, ADL and IADL limitations, and mortality; but a decreased likelihood of homecare use and hospital stay compared to older adults with cancer only or dementia only (some outcomes were not significant for dementia only group). Findings point out the risk of increased functional decline and mortality in older adults with comorbid cancer and dementia. Future research is needed to explore the contributing factors of the risk and identify interventions to promote physical function and reduce mortality for this population.

